# How public catering accelerates sustainability: a German case study

**DOI:** 10.1007/s11625-022-01183-2

**Published:** 2022-08-23

**Authors:** Melanie Speck, Lynn Wagner, Felix Buchborn, Fara Steinmeier, Silke Friedrich, Nina Langen

**Affiliations:** 1 Wuppertal Institute for Climate, Environment and Energy gGmbH, Doeppersberg 19, 42103 Wuppertal, Germany; 2 Faculty of Agricultural Sciences and Landscape Architecture, Osnabrueck University of Applied Science, Am Kruempel 31, 49090 Osnabrueck, Germany; 3 Institute for Sustainable Nutrition (iSuN), Muenster University of Applied Science, Corrensstr. 25, 48149 Muenster, Germany; 4 Institute of Vocational Education and Work Studies, Department Education for Sustainable Nutrition and Food Science, Technical University of Berlin, Marchstraße 23, 10587 Berlin, Germany

**Keywords:** Business catering, Sustainable nutrition, Sustainable diet, Nutritional footprint, Carbon footprint, Material footprint

## Abstract

Public catering has become increasingly important in recent years. With increasing annual customers, the sector’s impact on the environment is also growing continuously. At the same time, public catering offers a lever to promote sustainable nutrition that has rarely been used so far. Small changes in kitchen practices and food offers can thus be multiplied into a significant positive impact on environmental challenges, such as climate change or loss of biodiversity due to the large number of servings. In contrast to private households, management decisions in public catering can influence the food-related environmental impact of thousands of customers. This article deals with the nationwide level of greenhouse gas (GHG) emissions and resource use in the German public catering segment “business” and its saving potentials by different scenarios of unsupported and supported recipe revision. In this paper, we define "unsupported" as the intuitive optimization of recipes by employees of public catering businesses. In contrast, “supported” approaches had to meet specific target goals, for example of the Deutsche Gesellschaft für Ernährung; engl. German Nutrition Society or the sustainable level. Specifically, we will test how (A) an unsupported recipe revision, (B) a recipe revision based on dietary recommendations and (C) a recipe revision using scientific guidance affect the environmental impact of a dish. As a methodological framework, an online survey of public catering companies was conducted as well as a scenario analysis at menu level and at nationwide level. The results are based on empirical data on the one hand, and on extrapolations on the other. The results show that the nationwide implementation of recipe revision according to scientific guidance—such as concrete target goals for the GHG emissions per serving—can save up to 44% of resource use in the German business catering sector (which corresponds to 3.4 million tons of resources per year) and as much as 40% of GHG emissions (0.6 million tons GHG emissions per year). Even in the scenario of unsupported recipe revision, GHG and resource savings of up to 20% can be realized. The results show that public catering can reduce its material and carbon footprint by 20% overnight. Moreover, the findings show indications for the sustainable transformation of public catering. Nevertheless, it must be noted that these are some first steps of the transformation, which will require further changes with even greater impacts and political activities.

**Supplementary Information:**

The online version contains supplementary material available at 10.1007/s11625-022-01183-2.

## Introduction

Encouraging people to adopt a more sustainable diet is one important step for a sustainable society. Changing dietary habits can significantly contribute to mitigating negative environmental impacts such as climate change or the loss of biodiversity, for example, through a predominantly plant-based diet, the consumption of organic food or the reduction of food waste (Campbell-Arvai et al. 2012; Hoolohan et al. 2013; Godfray et al. 2018; Poore and Nemecek 2018; Abrahamse 2020). At the same time, a sustainable diet does not only affect environmental aspects, but can also improve individual health (Lukas et al. 2016; Willett et al. 2019; Strid et al. 2021). The simultaneous consideration of environment and health is also favored by the current state of knowledge, as both dimensions require similar actions (Tilman and Clark 2014; Speck et al. 2018; Chai et al. 2019). To meet the needs of ecosystems, livestock and health, consumption guidelines for individuals have been suggested at approximately 2 tons of resources and 0.35 tons of CO_2_-eq per year on food (Lettenmeier et al. 2014; Lukas et al. 2016; Speck et al. 2021). This corresponds to an environmental budget of 600–800 g CO_2_-eq per main meal (Lukas et al. 2016; Speck et al. 2021). For comparison, around 1.63 tons of greenhouse gases are currently emitted per capita and year (UBA 2015). Therefore, the target value is far exceeded. Furthermore, it cannot be dismissed that this target value will have to be corrected downward in future as a result of less savings from other industrial sectors. However, nutrition and consumption activities are quite complex and difficult to change—especially when they take place in private households (Nestle et al. 1998; Hummel and Hoffmann 2016; Matthies 2018; Abrahamse 2020). Against this background, public catering should be brought into focus—for strategic and efficiency reasons. This sector can initiate a change in eating habits through small or large changes in its offerings, as long as they are tasty and accepted by customers.

The public catering sector includes catering in nurseries, schools and universities as well as business catering and catering in care facilities, e.g., hospitals (Pfefferle et al. 2021; Teitscheid et al. 2018). Whether it is a school canteen or a cafeteria at a workplace, a look at the current state of public catering offerings shows that neither health-promoting nor sustainable alternatives are very popular (Lassen et al. 2013). In 2018, currywurst and fries, and schnitzel and spaghetti bolognese were recently voted as the top five most popular midday meals in German business catering (Apetito 2019). Although these dishes always enjoy great popularity, they are not recommended in everyday life, from a health or from an environmental perspective (Cross et al. 2007; Tilman and Clark 2014; Goldfrey et al. 2018).

Against this background, there is a great potential for reducing environmental impacts such as greenhouse gas (GHG) emissions or the use of natural resources, especially in public catering, which includes business catering (Speck et al. 2020). This potential is reinforced by the sector’s enormous leverage effect resulting from its wide reach. For example, the importance of public catering has been increasing in Germany for years, if one disregards the COVID-19 pandemic. As the second largest sales channel of the German food industry, it serves about 12.4 billion customers annually—and the trend is rising (BVE 2019, 2020). Business catering has a significant share in this and accounts for around 1.6 billion meals per year (DEHOGA 2013; BVE 2020). Although the global COVID-19 pandemic is expected to cause a decline in sales figures in 2020 and 2021, a renewed increase is expected in the post-COVID-19 period.

One of the most important starting points for the transformation toward a more sustainable business in public catering is the optimization of recipes and menus in a more sustainable way. These form the basis for the kitchen staff's daily work and the offered menu. While in the past, vegetarian options were mainly created by omitting the meat component and were therefore primarily composed of side dishes, today there are a number of more modern ways of revising recipes and menus. The dynamic cooperation with public caterers within the NAHGAST project^1^ (Langen et al. 2022) has resulted in different approaches to revise their own food offerings at different levels of implementation. Table 1 summarizes the most relevant revisions from an environmental perspective. As can be seen in this table, revisions at the recipe level are less complex to implement. For example, the replacement of specific ingredients within an established recipe can be found at this level. Furthermore, the changes at menu level are more complex, for example, the development of new sustainable dishes. The most effort is associated with changes at the management level, since at this level, for example, the company's procurement channels need to be reorganized. In addition, there are a couple of principles that are not listed here, such as some principles of the Culinary Institute of America and the Harvard T.H. Chan School of Public Health. They invented a large range of principles, e.g., preferring whole-grain ingredients or buying local and seasonal food (The Culinary Institute of America and Harvard T.H. Chan School of Public Health 2019). However, in Table 1 we focus on revision types that are (1) tested within the NAHGAST project and (2) are associated with high savings in resource use or GHG emissions.

**Table 1:** Types of sustainable recipe revision in public catering (own illustration)

Level		Type of revision	Description	Complexity/time effort	Example
1	Recipe level	Ingredient substitution	Substituting components with high environmental or social impacts	Low	Substituting coconut oil with rapeseed oil
		Ingredient reduction	Reducing the size of components with high environmental or social impacts		Serving a smaller piece of chicken breast (70 g) instead of a bigger one (100 g)
2	Menu level	Dish replacement	Substituting a less sustainable dish in the menu	Medium	Substituting spaghetti bolognese by spaghetti with lentil sugo in the menu
		Product development	Creating a completely new recipe		Creating a new recipe, using lots of vegetables and smaller or no meat components, e.g., a cabbage pan with bacon
		Adjust frequencies	Dishes with a high environmental or social impact are offered less frequently within a 4-week menu. More sustainable dishes are increased in frequency	Low	Currywurst and fries will be offered instead of every Wednesday, only on the first Wednesday of the month
3	Management level	New procurement strategy	Align procurement to ensure a sustainable supply	High	Complement one full-range supplier with regional or more sustainable suppliers to have access to a diverse range of products and product qualities

This paper focuses on revisions at the recipe level, as these are less complex to implement, on the one hand, and still associated with a large leverage due to the high number of daily servings, on the other. Various studies estimate that optimizing recipes can save about 25% (Hoolohan et al. 2013; Scharp et al. 2019; Speck et al. 2020) of GHG emissions. Based on the billions of meals consumed annually in the public catering sector, the potential for a sustainable transformation is enormous.

The main topic of this paper is to consider different scenarios of supported and unsupported recipe revisions and to measure their impact on resource use and GHG emissions. Therefore, we will test how (A) an unsupported recipe revision, (B) a recipe revision based on dietary recommendations and (C) a recipe revision using scientific guidance affect the environmental impact of a dish. Thus, the following research question arises:

- **RQ1:** Which saving potentials for resource consumption and greenhouse gas emissions result from different unsupported and supported recipe revision scenarios?

In addition to the saving potential at menu level, the results will also be used to model the impact of the scenarios on GHG and resource use in the business catering sector on a nationwide level. Accordingly, the second research question is:

- **RQ2:** What environmental impacts can result from the nationwide implementation of unsupported and supported recipe revision strategies in business catering?

## Material and methods

### 2.1 Different strategies for the revision of recipes

For the revision of recipes and menus, there are different tools or guidance for use in practice to support a more sustainable offering. One approach is to implement qualitative and quantitative dietary recommendations or guidelines for the composition of dishes and menus, for example from the Planetary Health Diet (PHD) (Willett et al. 2019) or the DGE quality standard (DGE 2020). These provide recommendations for the frequency and quantity of product groups or ingredients and their implementation can reduce negative environmental impacts as well as improve an individual's health (Springmann et al. 2020). Based on the recommendations of the PHD or the DGE quality standards, animal-based products (egg, meat, fish, dairy products) should not exceed more than 25–30% of the daily food intake (Oberritter et al. 2013; DGE 2019; Willett et al. 2019).

In addition, online assessment tools, such as the NAHGAST online tool,^2^ can support the recipe revision by providing a scientific sustainability assessment of the recipes. The NAHGAST online tool assesses a midday meal's impact on the environment, nutritional and social issues. This tool was developed in the first phase of the NAHGAST project and is based on the *Nutritional Footprint* (Lukas et al. 2016). Indicators were selected for each of the dimensions and specific target values were defined to make those dimensions measurable and assessable. These target values are based on a boundary approach, the so-called sustainable level (SL) (Lukas et al. 2016), which are derived from the idea of the planetary boundaries recommended by Rockström et al. (Rockström et al. 2009; Steffen et al. 2015; Persson et al. 2022). Thus, the SLs approach works less on the composition of meals and more on achieving quantitative specifications. Table 2 contains an overview of all indicators and target values of the NAHGAST online tool. On the one hand, the SLs are based on scientific recommendations. For example, the SLs of the nutritional indicators are derived from the recommendations of the DGE. On the other hand, the indicators are based on target values, such as the environmental indicators. Table 2 also includes indicators and SLs for the economic dimension. They were not integrated into the final implementation of the NAHGAST online tool, but shown here for the sake of completeness.

**Table 2:** Indicators and sustainable levels applied in the NAHGAST online tool (modified, based on Lukas et al. 2016; Engelmann et al. 2018)

Dimension	Environment	Social	Nutrition	Economic
Indicator (SL per serving)	Material footprint (< 2670 g) Carbon footprint (< 800 g)	Share of fair ingredients (> 90%) Share of animal-based food that fosters animal welfare (> 60%)	Energy (< 670 kcal) Fat (< 24 g) Carbohydrates (< 90 g) Sugar (< 17 g) Fibers (> 8 g)	Popularity (without quantified target value) Cost recovery (without quantified target value)

**Fig. 1:**
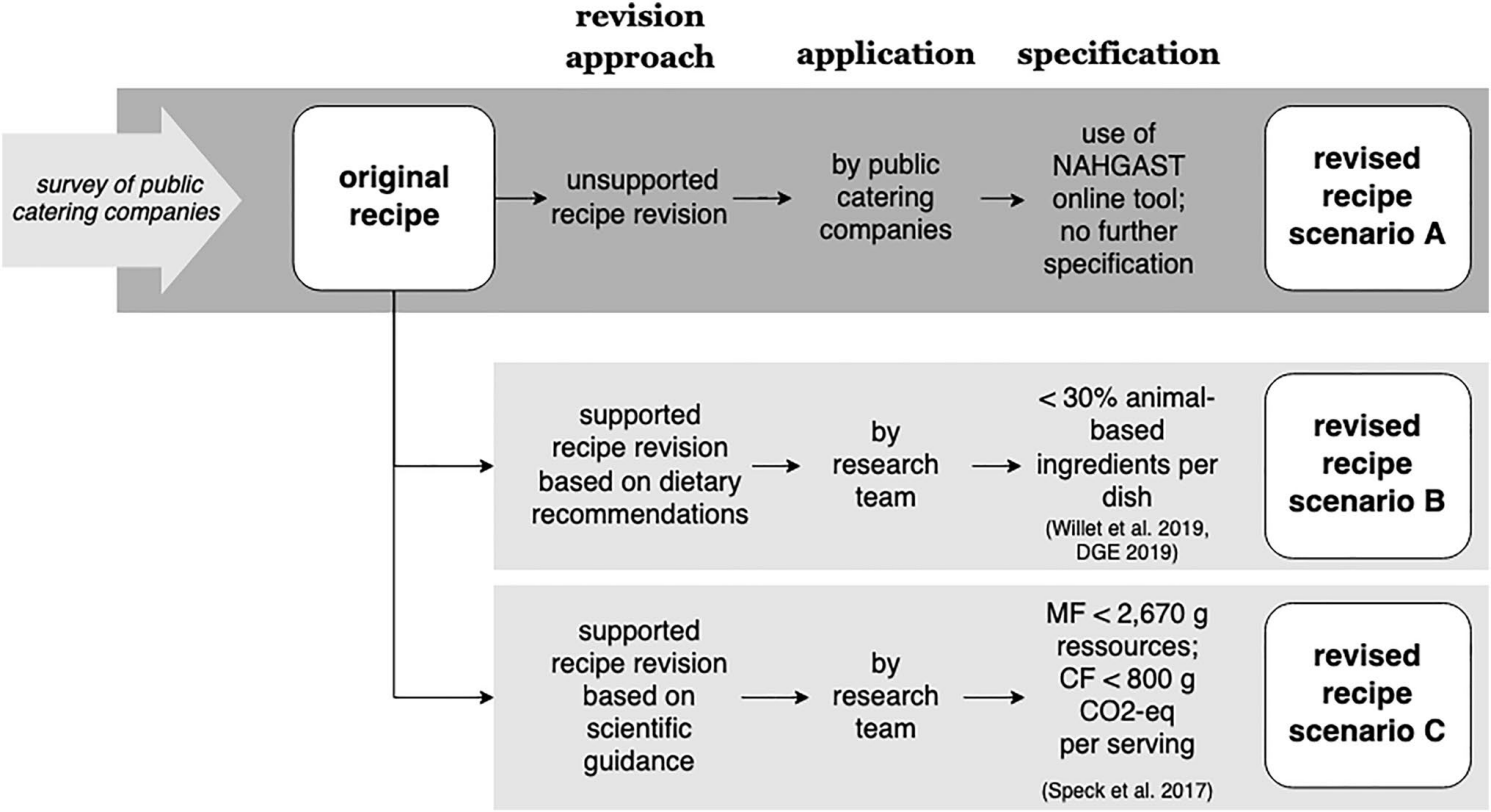
Methodical framework of the recipe revision scenarios

In this paper, we examine the impact of these tools, recommendations and guidance on the environmental and nutritional dimension of a midday meal. For this purpose, three different scenarios for optimizing public catering recipes were modeled and analyzed (see Fig. 1). Original recipes from public caterers were used as a reference for all scenarios.

Scenario A describes the unsupported revision of recipes by practitioners in public catering companies. These belong predominantly to company catering or catering in schools or universities. For each company, the survey was primarily completed by the persons responsible for menu planning. In this scenario, the practitioners revised their recipes using the NAHGAST online assessment tool, but without any further specifications. The original and revised recipes were collected in an online survey. In addition to the recipe, preparation methods were also surveyed.

Scenario B describes the recipe revision according to dietary recommendations for the use of animal-based products in the menu composition. In contrast to scenario A, the recipe revisions were modeled by the research team rather than conducted by practitioners. In this scenario, the revised recipe must meet the specification that the dish contains less than 30% animal-based products, according to the recommendations of PHD and DGE quality standards (DGE 2019; Willett et al. 2019). The following steps were taken to improve the recipes’ environmental footprints: first, those ingredients with the highest impact in terms of material footprint and carbon footprint were reduced. The general approach was as follows: as much as necessary, but as little as possible. In this way, we wanted to stay as close as possible to the original concept of the recipe. The high impact ingredients were replaced by less climate-intensive ingredients, to have the same portion size. When replacing ingredients, the focus was primarily on those ingredients for which adequate, less climate-intensive equivalents exist that fulfill the same function in the dish. For example, butter for frying was replaced by rapeseed oil. Cream was replaced by soy cuisine.

Scenario C describes the recipe revision according to scientific guidance for the carbon footprint and material footprint of a midday meal. More specifically, the recipe was revised so that the midday meal's material footprint was less than 2670 g resources per serving and its carbon footprint less than 800 g CO_2_-eq per serving, according to the SL (Speck et al. 2017). Similar to scenario B, the recipe revisions were modeled by the research team. The optimization steps were the same as in scenario B.

#### Survey of public catering companies

The original recipes as well as the optimized recipes according to scenario A were collected in an online survey conducted as part of the NAHGAST II project. In this way, it was ensured that the recipes were also relevant to practice and were actually offered in public catering.

For this purpose, a controlled online survey was conducted using the online survey tool SurveyMonkey. All public catering companies of the NAHGAST II project (*n* = 20) were given access to the survey. The survey was conducted in the period between July and September 2020. During this period, the survey was completed by eight companies. Collectively, this sample sells more than 100,000 midday meals per day and thus has a wide outreach. The questionnaire contained both open and closed questions on basic data about the company, the application of the NAHGAST online tool and a survey section for original recipes and variants that had been optimized using the NAHGAST online tool. With regard to the recipe collection, all ingredients (excluding ingredients in insignificant amounts like herbs or spices) and their quantities were captured, as well as the preparation method and duration. A total of 20 recipes (10 original recipes and 10 optimized variants) were completely transmitted and could be taken into account in the final results. Five of the original recipes were vegetarian recipes, and the remaining five contained meat.

#### Modelling recipe revisions based on dietary recommendations and environmental target values

The starting point for the recipe revision according to scenarios B and C are the original recipes from the survey.

For scenario B, these recipes were transferred to the Microsoft Excel spreadsheet program. Afterward, the composition of the recipes was modified in such a way that the proportion of animal products was less than 30%. For this purpose, on the one hand, weight proportions of ingredients were changed. On the other hand, ingredients were completely removed from the recipe and equivalent plant-based products were added instead. The total weight of the serving always remained constant. Recipes that already contained less than 30% of animal-based products before the revision were not revised.

For scenario C, the original recipes were first transferred to the NAHGAST online tool. Recipes whose material footprint was already equal to or smaller than 2.670 g resources per serving and corresponded to the carbon footprint target value of 800 g CO_2_-eq were not modified. The remaining recipes were revised until the target values were both fulfilled. As in scenario B, the weight of individual ingredients was changed or ingredients were completely removed or added. Again, the total weight of the serving was always kept constant.

### Assessment of the environmental impact per menu: RQ1

All recipe data (original recipes, recipes of scenario A, B and C) were transferred to the Life Cycle Assessment software openLCA to model the recipes’ GHG emissions and use of natural resources per serving. In total, we analyzed 70 ingredients. The methodology followed the standardized approach of the life cycle assessment according to DIN EN ISO 14040:2021-02 (2021) and DIN EN ISO 14044:2006-07 (2021). The use of natural resources is measured by the material footprint. This is a measurement for the life cycle resource use of a meal according to the MIPS concept (material input per service unit) (Schmidt-Bleek 1998; Liedtke et al. 2014). It comprises the direct and indirect demand for abiotic materials, which means all mineral raw materials, including economically unused raw materials, as well as biotic raw materials, which include mainly plant-based biomass from agriculture and forestry. The material footprint is expressed in kilograms of resources per kilogram of product. The carbon footprint is the total amount of emitted greenhouse gases and is expressed in kilograms of carbon dioxide equivalents caused directly and indirectly by an activity or released over the different life stages of a product, according to the IPCC 2007 methodology (IPCC 2008). The environmental data were taken from the Ecoinvent database version 3.1 and 3.6. A midday meal’s portion was defined as the functional unit in each case. The portion sizes range from about 230 to 600 g depending on the recipe and target group. These have been determined by the practitioners. The system boundaries include the agricultural production and further processing of ingredients, distribution processes up to the commercial kitchen as well as the preparation in the commercial kitchen. The LCA is based on an attributional approach. In the case of co-products, an economic allocation has been applied.

Based on the individual results of the recipes, an average value was also calculated. The difference between the result of the original recipe and the revised recipes from scenario A, B and C forms the absolute savings per portion, from which the relative saving potential can also be derived:

$$\mathrm{Absolute\,MF\,saving\,potential}={\mathrm{MF}}_{\mathrm{original\,recipe}}-{\mathrm{MF}}_{\mathrm{revised\,recipe }\left(\mathrm{A}\right)},$$

$$\mathrm{Relative\,MF\,saving\,potential}=\frac{100}{{\mathrm{MF}}_{\mathrm{original\,recipe}}}\times \left({\mathrm{MF}}_{\mathrm{original\,recipe}}-{\mathrm{MF}}_{\mathrm{revised\, recipe }\left(\mathrm{A}\right)}\right).$$

Moreover, the average resource use and average GHG emissions for the original and optimized dishes are determined using the following formula, shown here as an example for the average material footprint of the original recipes:

$$\mathrm{Average }{\mathrm{MF}}_{\mathrm{original\,recipes}}=\frac{{\mathrm{MF}}_{\mathrm{original\,recipe }\,1}+{\mathrm{MF}}_{\mathrm{original\,recipe }\,2}+\cdots +{\mathrm{MF}}_{\mathrm{original\,recipe }\,n}}{n}.$$

### Assessment of the nutritional impact per menu

In addition to the assessment of the environmental data, an analysis of nutritional values was conducted. For this purpose, the considered nutrients were analyzed for all recipes to be considered as original recipes as well as optimized recipes. The nutritional values were calculated using the data set of the German federal food catalog (Bundeslebensmittelschlüssel) version 3.02 published by the Ministry of Food and Agriculture (BMEL 2021). This comprises nutritional data for around 15,000 food products. In this analysis, we focused on the energy content and energy supplying nutrients (as well as salt), in detail: fat, fiber, carbohydrates, sugar and salt. The selection of the indicators has been taken from the concept of the NAHGAST online tool. Since the assessment includes only midday meals and does not take a full day's nutrition into consideration, a comprehensive analysis of macro and micro ingredients was omitted. Instead, the focus was on ingredients that are of particular importance for midday meals and can be used for the communication with customers, for example energy content. In addition, we did not include protein in the analysis, since the recommended amount of protein is significantly exceeded in most of the German population (NVS II 2008). An evaluation of further indicators would be useful, especially when considering weekly meal plans or daily intakes.

In the same way as described in Sect. 2.2, an average score was calculated for each scenario and indicator for all recipes. This simplifies a comparison of the development of nutritional values between the scenarios.

### 2.4 Assessment of the environmental impact in the nationwide business catering sector: RQ2

Supplementary to the saving potential at recipe level, the results were used to model the environmental effects of the recipe revisions on a nationwide level. For this purpose, a scenario analysis was conducted to examine how many GHG emissions and resources could be saved by a nationwide recipe revision using the presented scenarios A, B and C as an example. Approximately 1.6 million meals are served in business catering per year (DEHOGA 2013; BVE 2020). For each of the scenarios, two assumptions for the nationwide dissemination were made: a conservative assumption in which 50% of the annually offered meals in Germany are optimized according to scenario A, B or C, and an ideal dissemination in which all menus offered per year are optimized according to scenario A, B or C (see Table 3).

**Table 3:** Overview and description of the dissemination assumptions (taking the example of scenario A)

Dissemination scenario	Assumption
Status quo	All meals served per year in business catering are prepared according to original recipes (1.6 million meals)
Conservative dissemination	50% of the meals served per year in business catering are cooked according to recipes that have been optimized using the NAHGAST online tool (0.8 million meals). The remaining meals are prepared according to original recipes (0.8 million meals)
Ideal dissemination	All meals served per year in business catering are prepared according to recipes that have been optimized using the NAHGAST online tool (1.6 million meals)

With the conservative assumption, the average material footprint and carbon footprint of the original recipes were taken for 50% of the menus in business catering (0.8 million meals per year). For the remaining 50%, the average material footprint or carbon footprint of the revised recipes according to scenario A, B or C was taken (0.8 million meals per year). This results in the absolute resource use and GHG emissions of the business catering assuming a conservative nationwide dissemination:

$$\mathrm{Conservative\,dissemination}=\left(\mathrm{average }{\mathrm{\,MF}}_{\mathrm{original\,recipes}}\times \mathrm{800,000}\right)+\left(\mathrm{average }{\mathrm{\,MF}}_{\mathrm{revised\,recipes }\left(\mathrm{A}\right)}\times \mathrm{800,000}\right).$$

For the ideal dissemination, the average material footprint and carbon footprint of the optimized recipes according to scenarios A, B and C were assumed for 100% of the offered meals in business catering (1.6 million meals per year):

$$\mathrm{Ideal\,dissemination}=\mathrm{average }{\mathrm{\,MF}}_{\mathrm{revised\,recipes }(\mathrm{A})}\times \mathrm{1,600,000}.$$

As a reference for both scenarios, the status quo resource use and GHG emissions were used. For this purpose, the average material and carbon footprints of the original recipes were estimated for 100% of the menus in business catering (1.6 million meals per year):

$$\mathrm{Status \,quo }{\mathrm{\,MF}}_{\mathrm{business \,catering }}\mathrm{\,per \,year}=\mathrm{average }{\mathrm{\,MF}}_{\mathrm{original \,recipes}}\times \mathrm{1,600,000}.$$

## Results

### Saving potentials at recipe level

First, the survey results were used to determine the average impacts per original recipe—more precisely the status quo of GHG emissions and the use of natural resources per menu. Based on the sample, the average resource consumption is 4.00 kg of resources and 0.91 kg of CO_2_-eq per menu.

Regarding the use of natural resources, the status quo could be improved in all three scenarios (A, B and C) (see Table 4). In scenario A (unsupported revision using the NAHGAST tool), the average material footprint per meal is 3.18 kg, which amounts to a reduction of 20.4% compared to the status quo. For the recipe optimization in scenario B (revision by dietary recommendations for the use of animal-based products), the result is slightly better. The average material footprint per meal is 3.04 kg providing a saving potential of 24.0%. The greatest savings can be realized in scenario C (revision by scientific guidance on environmental target values). With a material footprint of 2.23 kg per meal and a saving potential of 44.3%, the savings are twice as high as in scenario A and B.

**Table 4:** Savings in carbon and material footprint at menu level for scenarios A, B and C

Scenario	Material footprint in kg resources/serving	Relative savings compared to status quo in %	Carbon footprint in kg CO_2_-eq/serving	Relative savings compared to status quo in %
Status quo	4.00	–	0.91	–
Scenario A: unsupported recipe revision	3.18	20.4	0.72	21.1
Scenario B: supported recipe revision based on dietary recommendation	3.04	24.0	0.71	21.3
Scenario C: supported recipe revision based on scientific guidance	2.23	44.3	0.55	39.6

Similar findings can be noted for the carbon footprint. Scenario A and B generate a carbon footprint of 0.72 and 0.71 kg CO_2_-eq per menu, which is almost the same level. The relative saving potential is 21.1% for scenario A and 21.3% for scenario B compared to the status quo of 0.91 kg CO_2_-eq. For scenario C, there is also greater saving potential for the GHG emissions. The average carbon footprint is 0.55 kg CO_2_-eq per menu, which corresponds to a saving potential of 39.6%.

Furthermore, when looking at the individual recipes at the ingredient level, it is possible to derive qualitative findings that illustrate differences between the approaches of the three scenarios. First of all, there are differences in the number of revised recipes. While in scenario A all dishes (10/10) were revised by the survey participants, in scenarios B and C only the recipes that did not meet the recommendations or target values were optimized. In scenario B, five out of 10 recipes were revised, in scenario C there were 6. In scenario A, even recipes that already have a very low impact were revised, such as a Mediterranean vegetable pan or a lentil sugo with spaghetti. Further on, there are differences in the selection of ingredients that are commonly substituted or reduced. While in scenario B and C mainly ingredients with a leverage effect were replaced, e.g., high-fat dairy products or meat products, in scenario A also ingredients with a low leverage were partially replaced, for example frozen vegetables. In addition, in scenario A, it appeared that the impact was reduced by smaller portion sizes. However, this measure is only useful if there is a lot of plate waste in the company. In the other scenarios, the portion size was kept constant. In scenario A and C, it can also be observed that the meat amounts were significantly reduced. In two of the meat-containing recipes, meat was completely removed. Although this leads to high savings in the carbon footprint and the material footprint, it can be associated with a loss of acceptance among customers of popular and traditional dishes. However, the character of each recipe was preserved as best as possible during the recipe revision. Thus, in the revision of the recipe spaghetti bolognese, the share of fresh vegetables was increased, while the amount of minced meat was partly reduced. In the rice pudding recipe, cow's milk was partly substituted by soy drink until the corresponding target values of the scenarios were met.

In summary, recipe revision offers the greatest saving potentials according to quantitative environmental target values. In terms of the material footprint and the carbon footprint, almost twice as high savings can be realized as in scenarios A and B. At the same time, the revision by environmental target values is also more complex in application. For example, the replacement of a meat component with a vegetable alternative enables great saving potentials, but can also lead to significant losses in customer acceptance if the revision is too substantial.

### Changes in nutritional values through recipe revision

The analysis of nutritional values has been primarily intended to show whether and, if so, in which way the considered nutrients of a recipe revision may differ.

Looking at Table 5, minor deviations result from the recipe revisions. The energy content decreases slightly with increasing complexity of the recipe revision. The energy content of the original recipes had an average of 460 kilocalories per serving, while the average of the revised recipes from scenario C is 430 kilocalories. The energy content decreases on average by about 6.5%. On the other hand, the fiber content increases significantly. In scenario C, the fiber content increases by about 17% compared to the original recipes. The fat content can be reduced by around one-fifth in all three optimization scenarios. At the same time, the carbohydrate content increases slightly. However, an increase in sugar in the optimized recipes can be ruled out. The sugar content remains largely constant. The same applies to the salt content.

**Table 5:** Changes in nutritional values per serving for the original recipes and optimized recipes in scenarios A, B and C

Scenario	Energy content in kcal per serving	Fiber in g per serving	Fat in g per serving	Carbohydrates in g per serving	Sugar in g per serving	Salt in g per serving
Status quo	460	7.2	18.5	45.8	10.8	1.2
Scenario A: unsupported recipe revision	452	8.6	15.9	53.3	10.3	1.1
Scenario B: supported recipe revision based on dietary recommendations	445	8.2	15.7	51.6	10.5	1.1
Scenario C: supported recipe revision based on scientific guidance	430	8.4	14.5	48.9	10.7	1.1

In summary, there was no deterioration in the considered energy supplying nutrients because of the recipe optimization. On the contrary, the fiber content was increased and the fat content significantly reduced.

### Saving potentials in the nationwide business catering

**Fig. 2:**
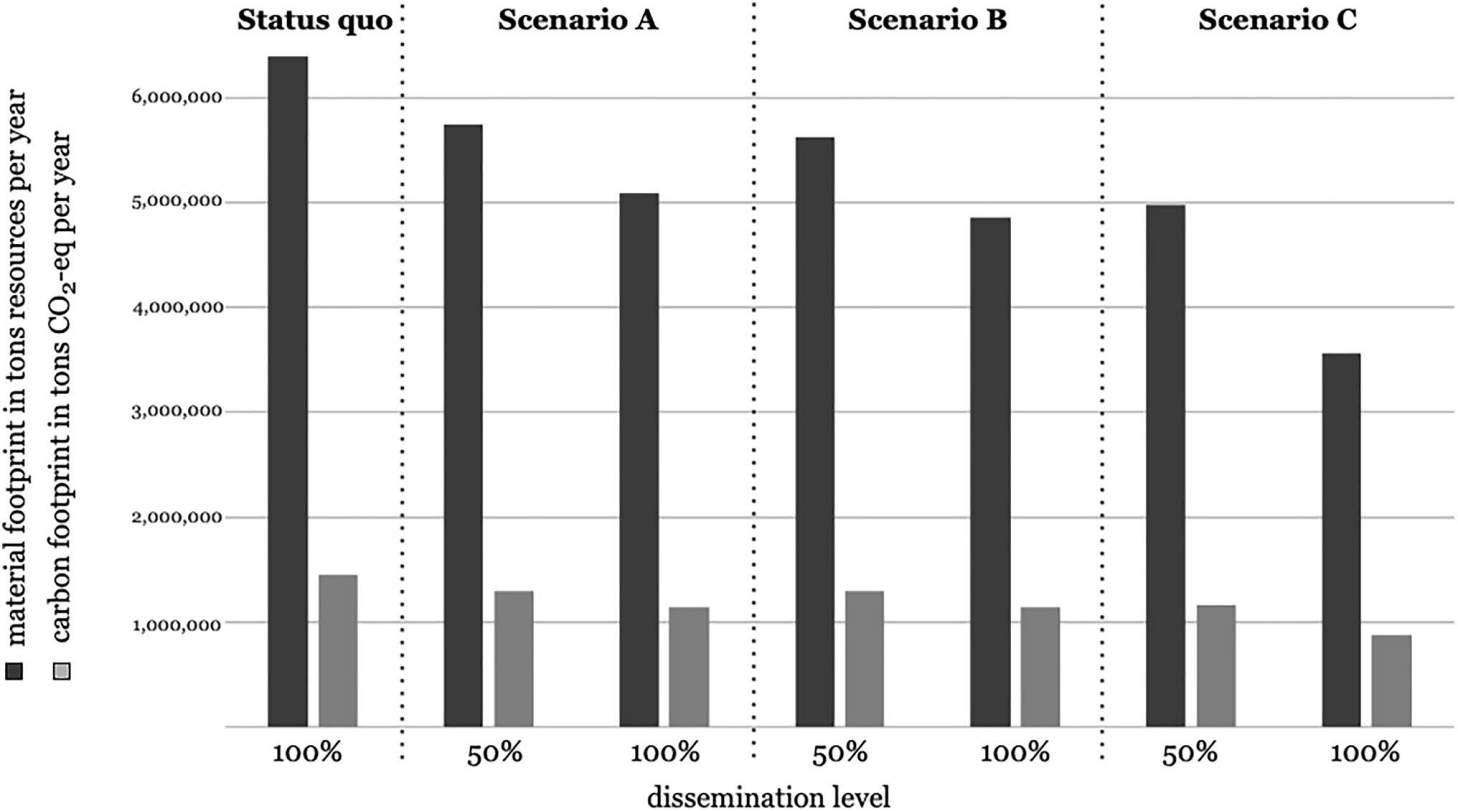
Savings in carbon and material footprint at nationwide level for scenarios A, B, and C and a conservative (50%) versus ideal (100%) dissemination

Based on the average impact per menu (derived from the original recipes) and the assumption of about 1.6 billion midday meals served in German business catering per year (DEHOGA 2013; BVE 2020), this results in an annual material footprint of 6.4 million tons for this sector (see Fig. 2). However, due to the sector’s large leverage effect, even in scenario A (unguided revision) and a conservative assumption on dissemination (50%), a remarkable amount of 0.65 million tons of natural resources can be saved. This corresponds to a relative saving potential of approximately 10%. Scenario B (revision by dietary recommendations for animal-based products) with conservative dissemination (50%) shows a slightly better result. Thus, 0.77 million tons of natural resources can be saved annually in this scenario. In the case of conservative dissemination (50%) of scenario C (revision by scientific guidance on environmental target values), 1.42 million tons of natural resources can be saved, which approximately corresponds to the saving potential of the ideal dissemination (100%) of scenarios A and B. The greatest savings can be achieved with ideal dissemination (100%) in scenario C. In this case, absolute savings of up to 2.83 million tons of resources are possible.

In terms of GHG emissions, the annual carbon footprint for business catering in Germany is 1.45 million tons CO_2_-eq. The nationwide savings in GHG emissions show a similar trend as the use of natural resources. While around 0.15 million tons of CO_2_-eq can be saved in scenario A and B assuming a conservative dissemination, scenario C offers savings of 0.29 million tons of CO_2_-eq. Again, the best result can be realized in scenario C with an ideal dissemination (100%) with savings of up to 0.57 million tons of CO_2_-eq.

In summary, it can be said that an unsupported recipe revision by the practitioners and/or the implementation of dietary recommendations can already make a significant impact on the reduction of GHG emissions and the use of natural resources in the field of business catering. However, twice as much can be saved if the target values of environmental indicators—such as material footprint and carbon footprint in this case—and their compliance is focused more stringently, as it is the case with nutritional indicators (e.g., energy content, fat content).

## Discussion

The paper aims to understand the status quo of the material footprint and carbon footprint of business catering in Germany and to identify saving potentials as well as differences in various recipe revision scenarios. Further, a nationwide perspective complements this research. Based on the findings, it was possible to illustrate the leverage effect of the business catering sector and to show various possibilities to use this leverage and to reduce the use of natural resources and GHG emissions. The greatest saving potentials could be realized by recipe revision based on clearly defined environmental target values and their achievement. Compared to the status quo, a saving potential of up to 44% with regard to the material footprint and 40% with regard to the carbon footprint can be realized. Almost half of the savings can be realized if the revision is done by the kitchen staff without any guidance or if the dish includes less than 30% animal-based products complying with the recommendation.

Against this background, the saving potentials from scenarios A and B correspond to the results of other studies, which estimate a saving potential of about 25% in the public catering sector by recipe revision (Hoolohan et al. 2013; Scharp et al. 2019; Speck et al. 2020). Although the results presented in this paper are based on a relatively small sample (*n* = 10 recipes), the results are likely applicable to other samples and settings.

Nevertheless, the recipe revision scenarios do not have an unlimited potential for widespread application. While the unguided optimization by the public catering companies enables savings of around 20% of the carbon footprint and material footprint, it must be noted that the recipe revision is comparatively inefficient. Recipes that do not require a revision (as they already have a low impact) were optimized and ingredients with minor or no positive effects were substituted. Thus, although this type of optimization is similarly effective as recipe revision based on dietary recommendations, it is less time efficient.

Furthermore, the saving potential determined in scenario C is significantly higher, but also associated with more significant revisions in the recipe composition. Considering this, it can be assumed that the dishes will not receive the same acceptance as the other revised recipes. The compliance with the target values only allows very small amounts of meat and dairy products, so that in some cases only 20 g of meat per serving remained after the recipe revision. Theoretically, this would still be feasible for minced meat, but it is hardly feasible for meals such as schnitzel. Either way, for the customers, there is a large discrepancy between the expected dish and the received one, which can lead to a loss of acceptance (Macdiarmid et al. 2016; Lorenz-Walther and Langen 2020). These results show that revision at recipe level is probably limited in practical implementation. At this point, it is more appropriate to consider menu revisions by developing new, more sustainable dishes that are less associated with traditional expectations among guests and kitchen staff.

Due to the COVID-19 pandemic, revised recipes could not be adequately tested and evaluated in public catering companies. Accordingly, we are unable to make valid conclusions about the acceptance of the revised recipes. However, for the revised recipes in scenario A, it can be stated that the practitioners know the preferences, wishes and expectations of their guests quite well. Their experience allows them to revise the dishes in such a way that they fit into the regular menu and are accepted by the guests. Acceptance is one of the most important success factors for the implementation of environmentally compatible and healthy food offerings and needs to be evaluated in more detail in upcoming studies. Garnett et al. (2019) also show that a more sustainable dish can be accepted by guests if the offering is slowly changed. In a large-scale study, the share of vegetarian dishes in the menu was doubled step by step, which led to a decrease in consumption of meat dishes by about 15 percentage points (Garnett et al. 2019).

From a nutritional perspective, all recipe optimization scenarios do not represent a deterioration in the considered nutritional values. On the contrary, the fiber content increases significantly and the fat content per portion decreases. However, to keep focusing on the development of micronutrients, further studies should not only focus on individual recipes of a kitchen, but also on entire weekly meal plans. In a further analysis, it could also be useful to include a more specific analysis of protein. As part of such a study, a change in the protein content and changes in the biological quality of the proteins should be focused on. Especially when replacing animal protein sources with plant-based ones, this could provide additional results.

Finally, it should be noted that the survey of the original recipes took place in summer. Although we assume that there are only minor interseasonal deviations, we cannot exclude the possibility of seasonal bias. To eliminate these, the survey period could be extended to 12 months in further research.

## Conclusion

As a conclusion, recipe optimization according to scientific guidance on environmental target values is a more effective way of recipe revision to reduce resource use and GHG emissions than the other considered approaches. Supporting materials such as the NAHGAST online assessment tool can support public catering companies in this context by providing a low-threshold sustainability assessment of menus. However, this process could also be simplified by the continuous inclusion of environmental data in inventory management systems—comparable to nutritional values (e.g., energy content). As a second point, it can be stated that an optimization of existing recipes is probably limited by the customers' expectations of the dish. Therefore, in addition to the optimization of established dishes, the focus should also be on the development of new dishes that are not associated with traditional expectations and that also meet the requirements of a sustainable diet. From a nutritional perspective, all recipe optimization scenarios do not represent a deterioration in the considered energy supplying nutrients, but it remains to be seen how the recipe revisions will affect other nutritional indicators, such as the biological quality of proteins.

Nevertheless, an unguided optimization or a recipe revision according to dietary recommendations such as the DGE quality standards or the PHD (DGE 2019; Willett et al. 2019) can also have a major impact. If all annually served menus in business catering would be prepared according to recipes that have been optimized by the public catering companies themselves, approximately 1.3 million tons of resources and 0.3 million tons of GHG emissions could be saved per year in business catering.

All in all, it can be said that the steps implemented within the framework show (1) good indications for the transformation of the sector, (2) that real-world lab approaches can be effective in practical implementation (Wanner et al. 2021) and (3) that the initiated changes represent just a beginning from a scientific point of view. The changes initiated in this research are already having an effect, but are still located in the "comfort zone". In the short to medium term, it must be able to implement more resource-effective changes in commercial kitchens (e.g., further expansion of vegetarian or vegan offerings, offer restrictions). This may ultimately lead to conflicting goals, which must then be recognized and addressed. Furthermore, these activities must also be politically anchored to address a societal transformation as soon as possible.

### Electronic supplementary material

Below is the link to the electronic supplementary material.

Supplementary file1 (XLSX 21 KB)

## Appendix

See Tables 6, 7.

**Table 6:** Savings in material footprint at nationwide level for scenarios A, B and C and a conservative versus ideal dissemination

Optimization scenario	Dissemination level	Material footprint per year (t/a)	Absolute saving potential (t/a)	Relative saving potential (%)
Status quo	–	6,395,302	–	–
Scenario A: unsupported recipe revision	Conservative (50%)	5,743,295	652,007	10.20
	Ideal (100%)	5,091,288	1,304,014	20.39
Scenario B: supported recipe revision based on dietary recommendations	Conservative (50%)	5,628,902	766,400	11.98
	Ideal (100%)	4,862,501	1,532,800	23.97
Scenario C: supported recipe revision based on scientific guidance	Conservative (50%)	4,980,102	1,415,200	22.13
	Ideal (100%)	3,564,901	2,830,400	44.26

**Table 7:** Savings in carbon footprint at nationwide level for scenarios A, B and C and a conservative versus ideal dissemination

Optimization scenario	Dissemination level	Carbon footprint per year (t CO_2_-eq/a)	Absolute saving potential (t CO_2_-eq/a)	Relative saving potential (%)
Status quo	–	1,449,620	–	–
Scenario A: unsupported recipe revision	Conservative (50%)	1,296,819	152,801	10.54
	Ideal (100%)	1,144,018	305,602	21.08
Scenario B: supported recipe revision based on dietary recommendations	Conservative (50%)	1,295,389	154,231	10.64
	Ideal (100%)	1,141,159	308,462	21,28
Scenario C: supported recipe revision based on scientific guidance	Conservative (50%)	1,162,736	286,884	19.79
	Ideal (100%)	875,851	573,769	39.58

## Footnotes

1 NAHGAST I and the subsequent project NAHGAST II (03/2015–05/2021) were funded by the German Federal Ministry of Education and Research and aimed at the initiation and dissemination of transformation processes for sustainable production and consumption in public catering. In addition to the transdisciplinary research team, more than 20 practical partners from different sectors of public catering (care, business, education) have been involved.

2 The NAHGAST online tool was developed within the research project NAHGAST I and published in March 2018. During the follow-up project NAGAST II, the calculator was updated, extended and evaluated in close interaction with more than 20 public catering companies.
